# Supplementary material for: Septal wall synthesis is sufficient to change ameba-like cells into uniform oval-shaped cells in Escherichia coli L-forms
Source: Commun Biol. 2024 Nov 26;7:1569. doi: 10.1038/s42003-024-07279-y (PMC11589767; doi:10.1038/s42003-024-07279-y)
Supplement: Supplementary file 1 — Supplementary Information [file 42003_2024_7279_MOESM1_ESM.pdf]

Septal wall synthesis is sufficient to change amoeba-like cells into uniform oval-shaped cells in *Escherichia coli* L-forms.

Masafumi Hayashi[1†], Chigusa Takaoka[1], Koichi Higashi[2], Ken Kurokawa[2], William Margolin[3], Taku Oshima[4*], Daisuke Shiomi[1*]

[1]Rikkyo University, Tokyo, Japan
[2]National Institute of Genetics, Shizuoka, Japan
[3] UTHealth-Houston, Houston, USA
[4]Toyama Prefectural University, Toyama, Japan
[†]Present address: Gakushuin University, Tokyo, Japan.

*** Correspondence:**
Taku Oshima: taku@pu-toyama.ac.jp
Daisuke Shiomi: dshiomi@rikkyo.ac.jp

Keywords: L-form, FtsZ, peptidoglycan, cell shape

Running title: Cell shape determination by FtsZ and septal cell wall.

a

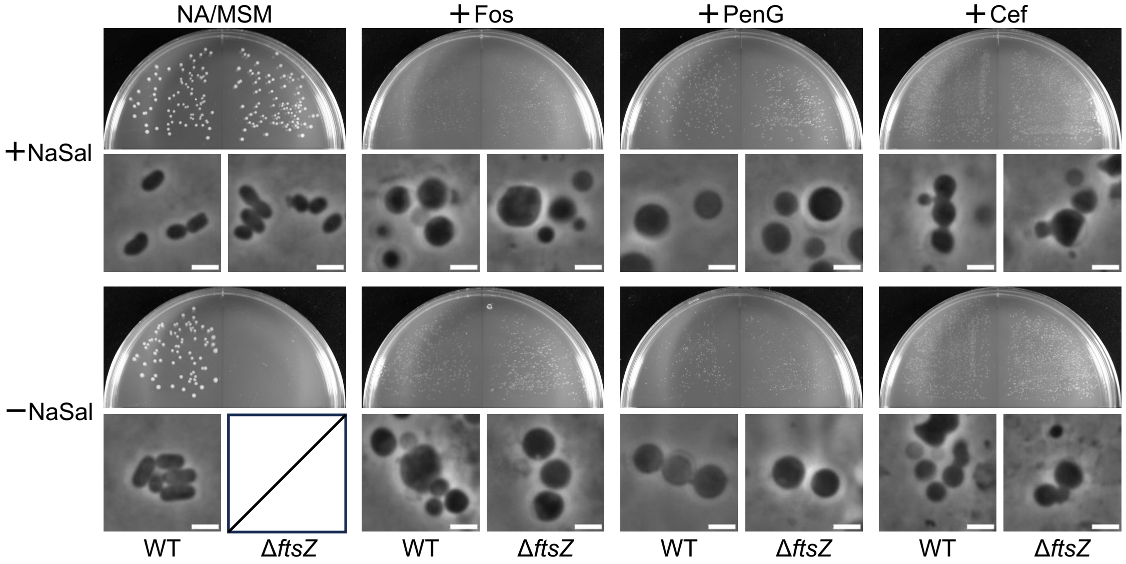

b

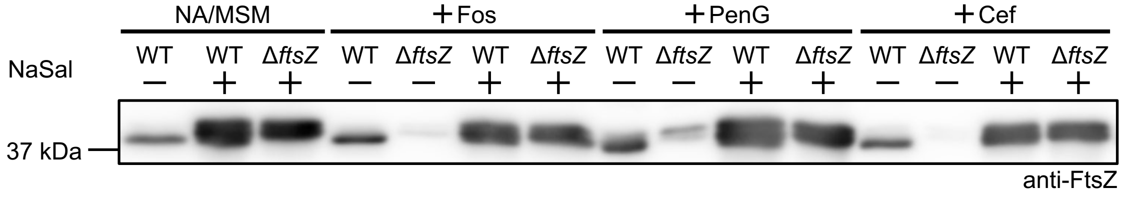

Supplementary Fig. 1. Conversion to L-form and FtsZ expression levels in Δ*ftsZ* cells.

(a). Colony formation of BW25113 or Δ*ftsZ* (RU2055: Δ*ftsZ::kan*) carrying pWM2765 encoding *ftsZ* in the presence or absence of NaSal with or without antibiotics (Fos, PenG, Cef). Plates were incubated for 3 days at 30° C under anaerobic conditions. Phase-contrast images of cells from a typical colony were shown in the inset. Scale bar: 2 μm.

(b). Immunoblotting of FtsZ protein. Samples were separated by SDS-polyacrylamide gel electrophoresis followed by immunoblotting with anti-FtsZ antibody.

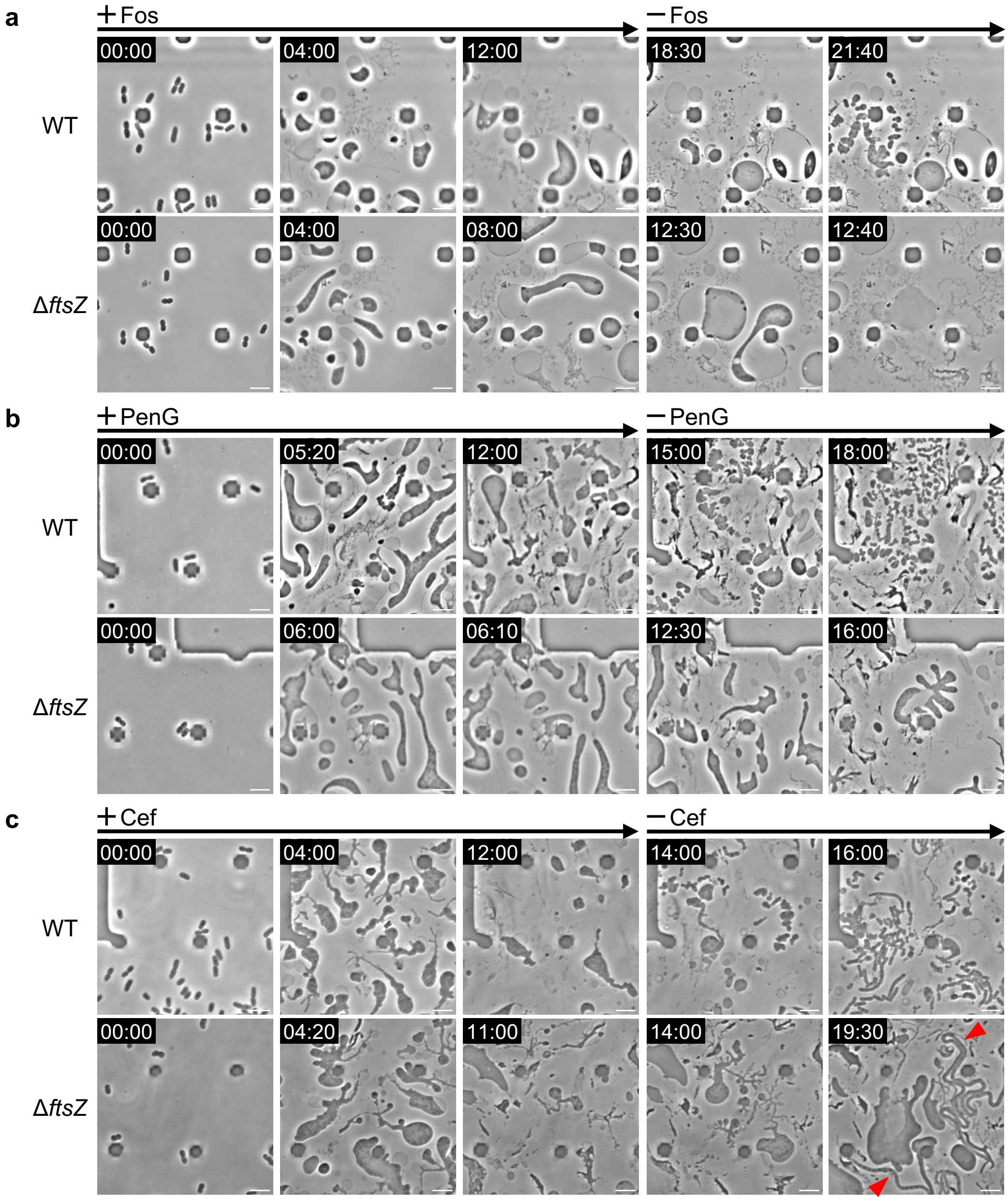

Supplementary Fig. 2. Conversion and reversal between L-form and walled cells in Δ*ftsZ* cells.
(a-c). Time-lapse images of WT (BW25113) and Δ*ftsZ* (RU2055) cells. Cells were grown in NB/MSM medium containing Fos (a), PenG (b) or Cef (c) under anaerobic conditions in the absence of NaSal. Images were taken every 10 min. The dark square-like structures are braces for the plate of the microfluidic device. Red arrows indicates abnormally long cells. Time in each picture is shown as hour: min. Scale bar: 5 μm. The height of the ceiling is 0.7 μm.

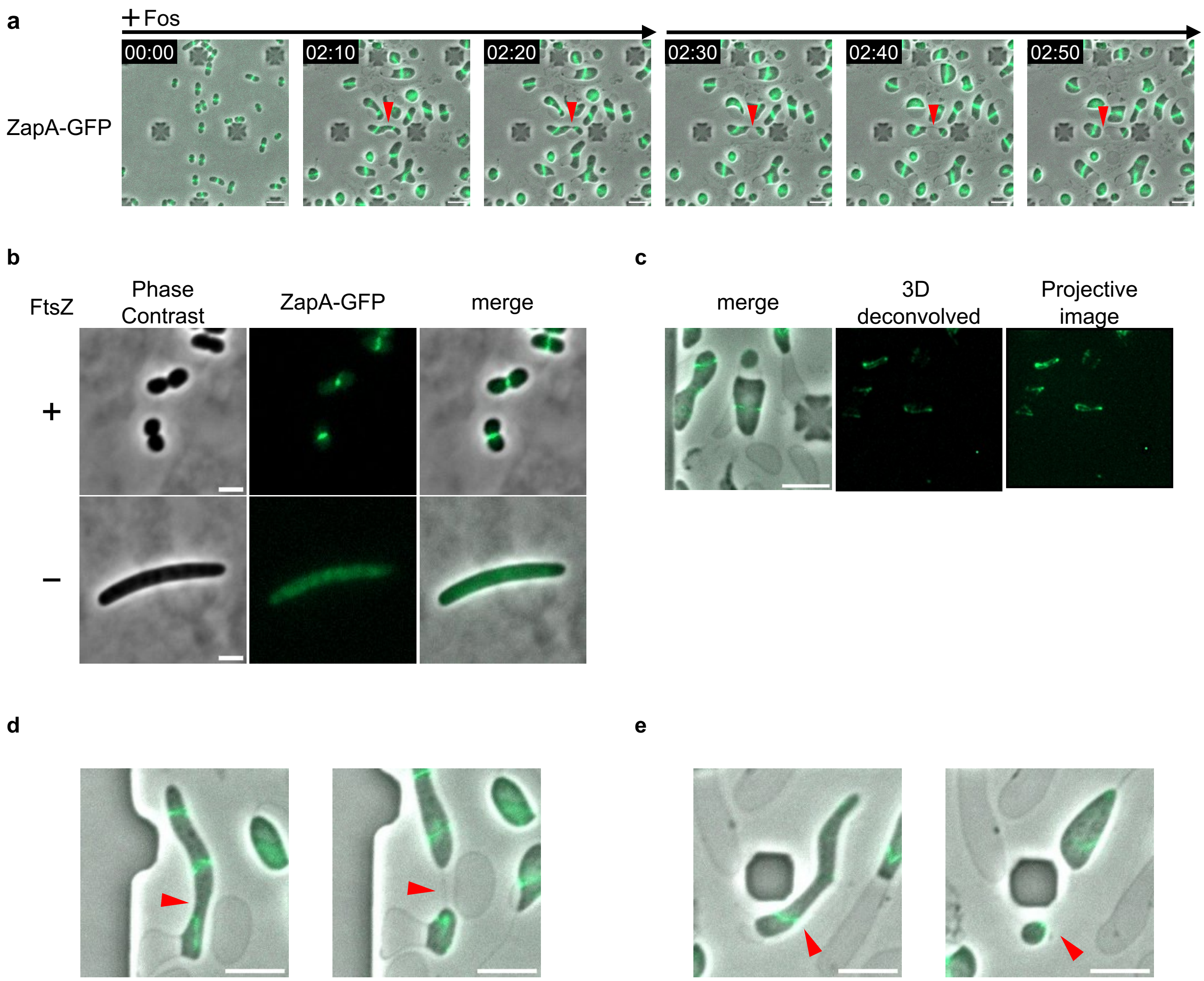

Supplementary Fig. 3. Subcellular localization of ZapA-GFP in L-form cells
(a) Time-lapse Images of RU1125 (*zapA-sfGFP::cat*) cells. Cells were grown in NB/MSM medium containing Fos. After the cell loading using NB/MSM medium containing Fos, the flow rate was set to 0 µL/hr. Red allows indicate cell division site.
(b) Images of RU2057 (*zapA-sfGFP ΔftsZ::Kan*) cells carrying pWM2765. Cells were grown to log phase in NB/MSM medium under aerobic conditions and mounted on a 2% agarose in M9 medium. Expression of *ftsZ* was induced by 10 µM NaSal. Scale bar: 2 µm.
(c-e) Images of RU1125 (*zapA-sfGFP::cat*) cells. Cells were grown in NB/MSM medium under anaerobic conditions containing Fos. After 4h, L-form cells were observed using the Z-stack function. Images were captured every 0.2 µm (c). Cells were grown in NB/MSM medium containing Fos. Red arrows indicate cell division site (d, e). Scale bar: 5 µm.

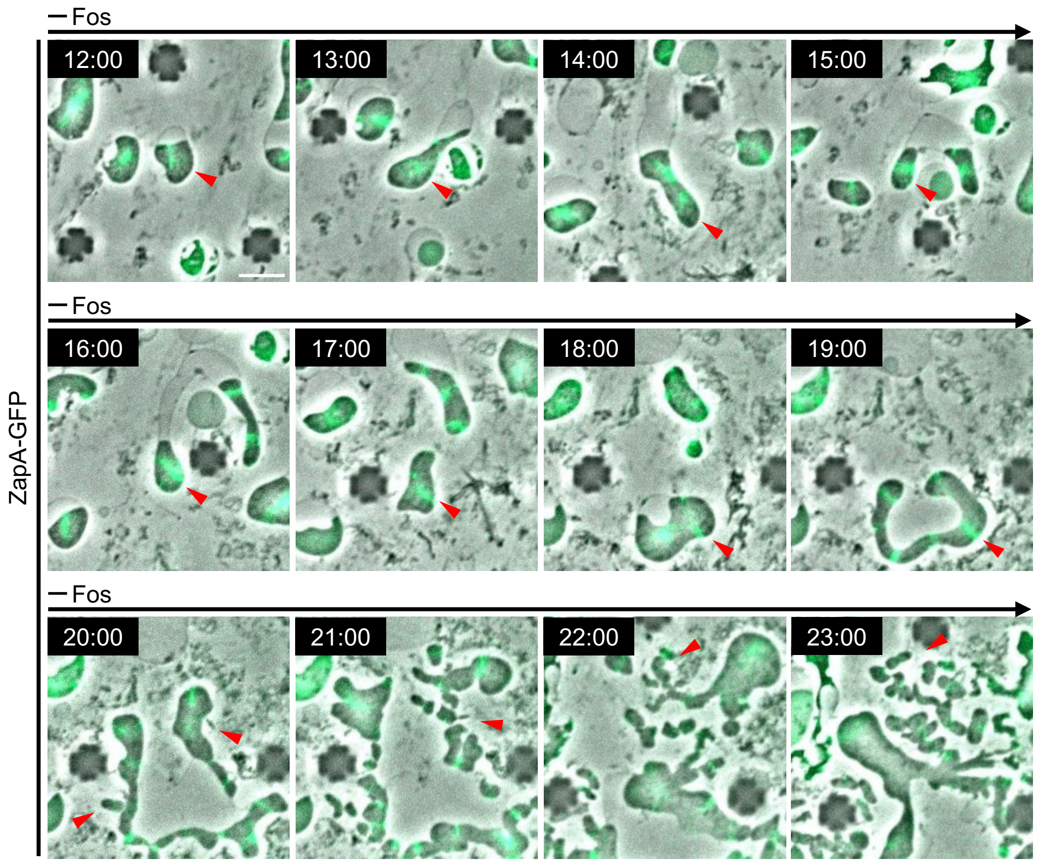

Supplementary Fig. 4. The Z ring persisted during the conversion to walled cell from L-from cells.
Time-lapse images of RU1125 (*zapA-sfGFP::cat*) cells. Cells were grown in NB/MSM medium containing Fos under anaerobic conditions. After 12 h, Fos was removed from the NB/MSM medium. Images were taken every 10 min. Red allows indicate one cell converting from L-form to walled cell. Scale bar: 5 μm.

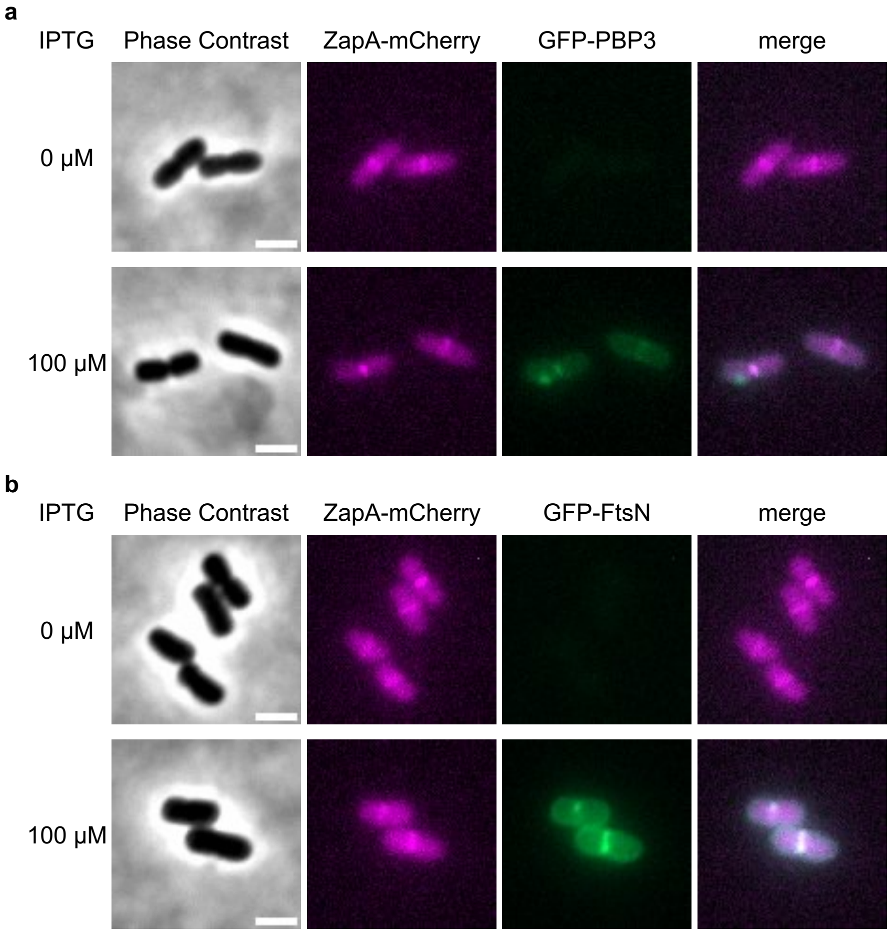

Supplementary Fig. 5. Subcellular localization of the late division proteins and ZapA-mCherry in walled cells.
(a, b) Images of RU2452 (*zapA-mCherry*) cells carrying pRU2282 (a) or pRU2281 (b). Cells were grown to log phase in NB/MSM medium under aerobic conditions and mounted on a 2% agarose in M9 medium. Expression of GFP-PBP3 (a) or GFP-FtsN (b) was induced by 100 μM IPTG. Scale bar: 2 μm.

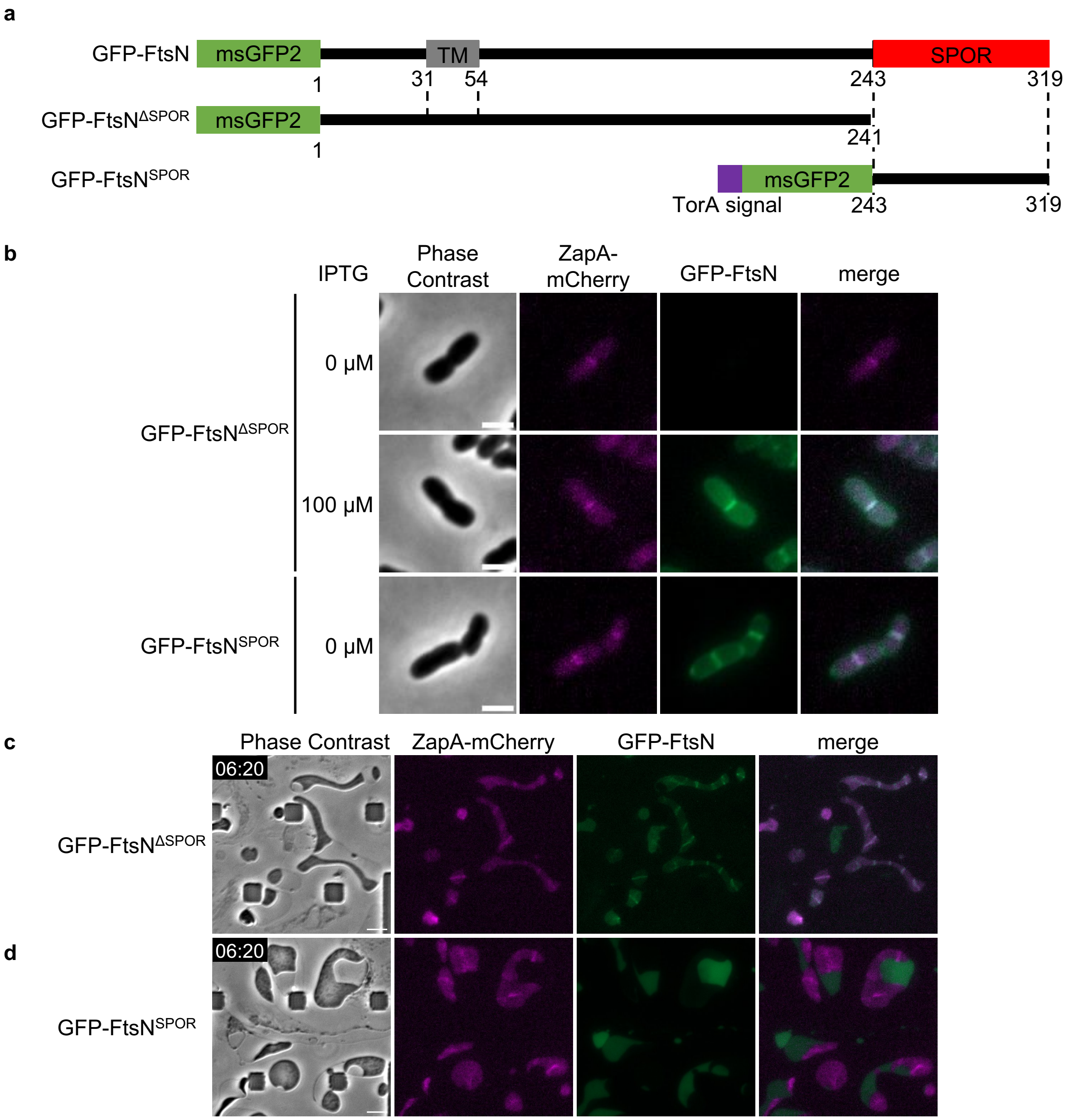

Supplementary Fig. 6. Subcellular localization of FtsN SPOR mutants in L-form cells.
(a) Schematic representation of FtsN protein which has a transmembrane domain (TM) and a C-terminal SPOR domain. FtsN and FtsN$^{\Delta SPOR}$ were fused msGFP2. FtsN^{SPOR} was fused with msGFP2 and a signal sequence of TorA (TorA^{ss}).
(b-d) Images of RU2452 (*zapA-mCherry*) cells producing FtsN mutants. GFP-FtsN$^{\Delta SPOR}$ was produced in the presence of 100 μM IPTG while GFP-FtsN^{SPOR} was produced without IPTG. Cells were grown in NB/MSM medium under aerobic conditions and mounted on a 2% agarose in M9 medium (b). Cells were grown in NB/MSM medium under anaerobic conditions containing Fos. After conversion to the L-form, the growth conditions were changed from anaerobic (c, d). Scale bar: 5 μm.

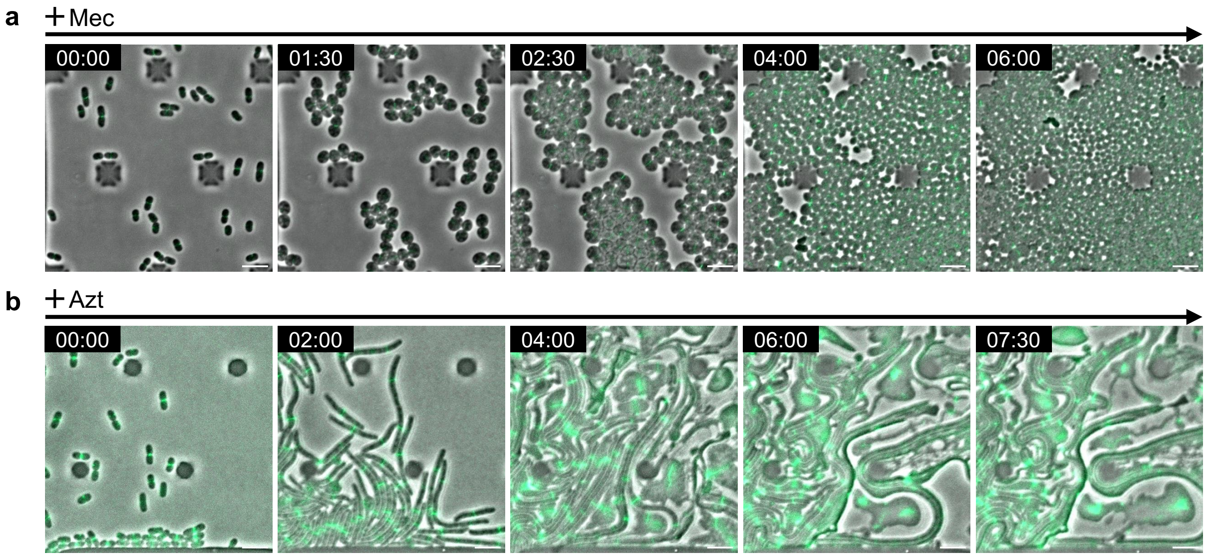

Supplementary Fig. 7. Subcellular localization of ZapA-GFP cells with Mec or Azt.
Time-lapse images of RU1125 (*zapA-sfGFP::cat*) cells. Cells were grown in NB/MSM medium containing Mec or Azt under anaerobic conditions. Scale bar: 5 μm.

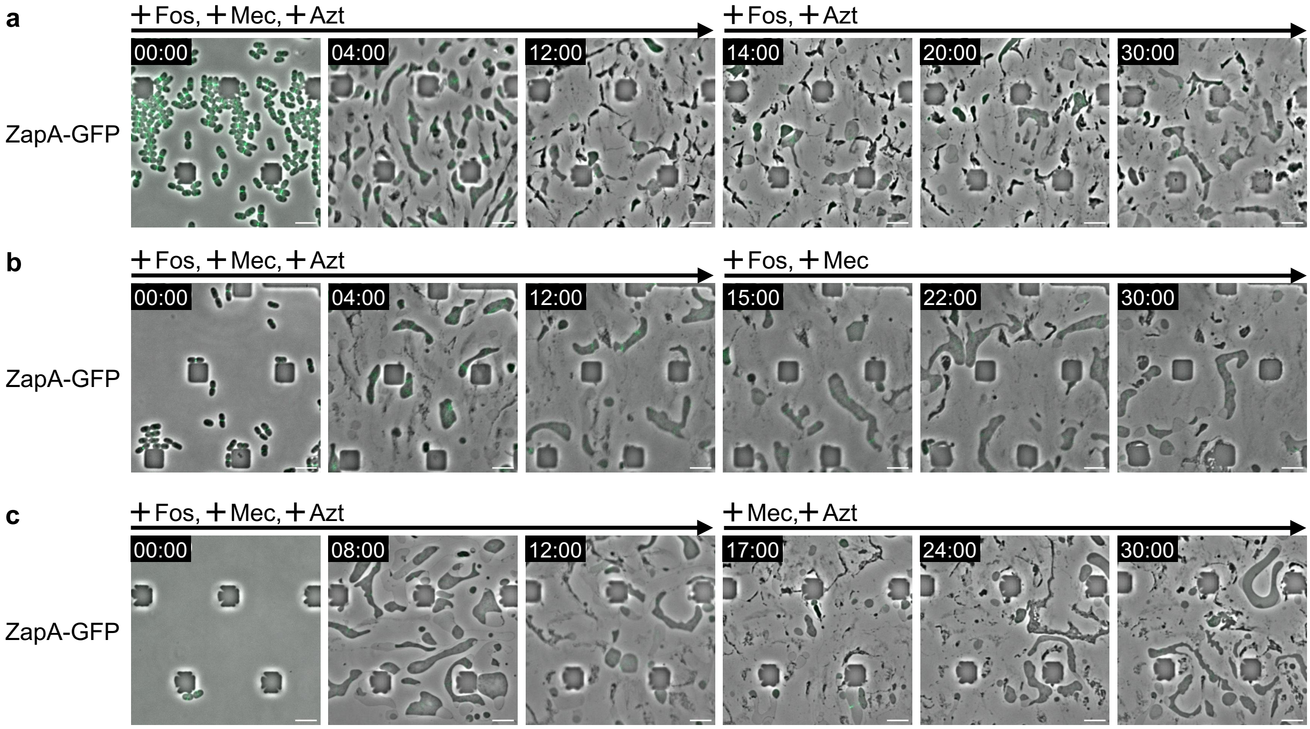

Supplementary Fig. 8. Conversion to L-form with Fos, Mec and Azt.
Time-lapse images of RU1125 (*zapA-sfGFP::cat*) cells. Cells were grown in NB/MSM medium containing Fos, Mec and Azt under anaerobic conditions. After 12h, Mec (a), Azt (b) or Fos (c) were removed from NB/MSM medium. Scale bar: 5 μm.

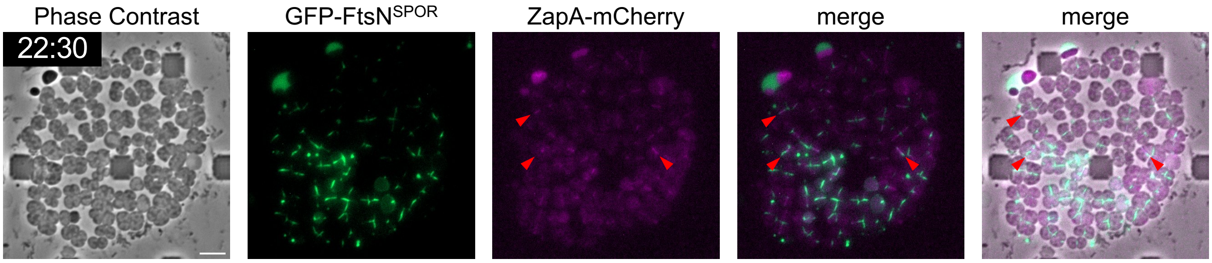

Supplementary Fig. 9. Subcellular localization of GFP-FtsN^{SPOR} in SWD cells
Images of RU2452 (*zapA-mCherry*) cells carrying a plasmid encoding GFP-FtsN^{SPOR} (pRU2624) are shown. Cells were grown in NB/MSM medium containing Fos, Mec and Azt. After 12 h, Fos and Azt were removed from the NB/MSM medium. After 16h, the growth conditions were changed from anaerobic to aerobic to observe ZapA-mCherry. Red arrows indicate typical cells showing colocalizations of GFP-FtsN^{SPOR} and ZapA-mCherry. Scale bar: 5 μm.

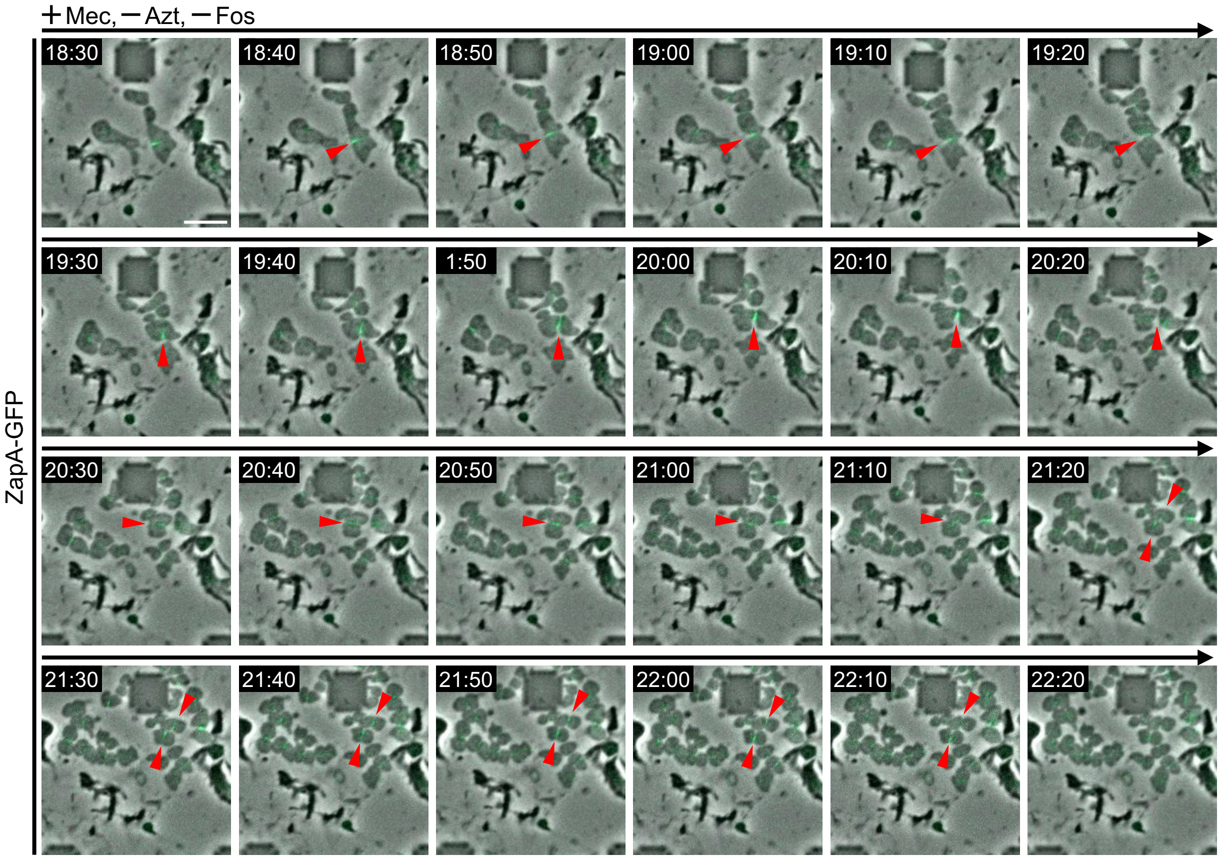

Supplementary Fig. 10. Subcellular localization of ZapA in SWD cells

Time-lapse images of RU1125 (*zapA-sfGFP::cat*) cells. Cells were grown in NB/MSM medium containing Fos, Mec and Azt under anaerobic conditions. After 12h, Fos and Azt were removed from NB/MSM medium. Red arrows indicate typical cell division sites. Scale bar: 5 µm.

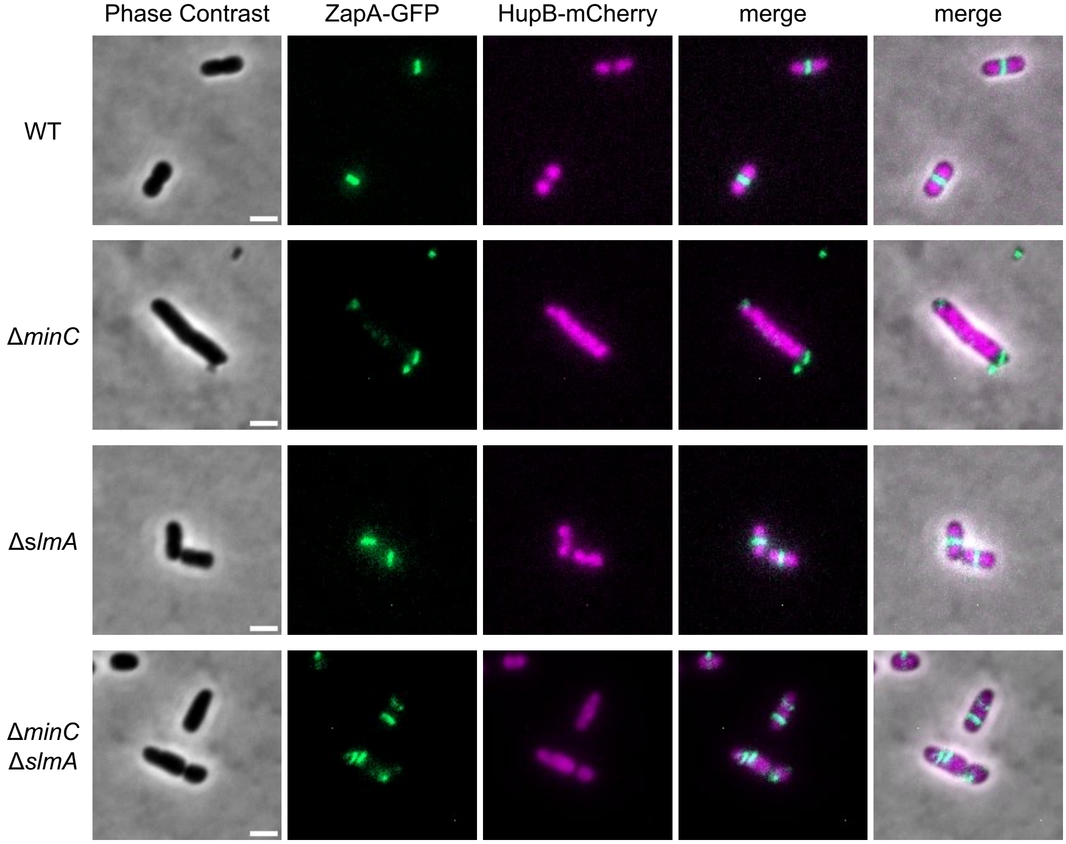

Supplementary Fig. 11. ZapA-GFP and HupB-mCherry in Z-ring position determinant deficient cells.
Subcellular localization of ZapA-GFP and HupB-mCherry in rod-shaped cells. RU1974 (WT), RU2401 (Δ*slmA*), RU2400 (Δ*minC*), and RU2409 (Δ*minC* Δ*slmA*) were grown to log phase in NB/MSM medium under aerobic conditions and mounted on a 2% agarose in M9 medium. Scale bar: 2 μm.

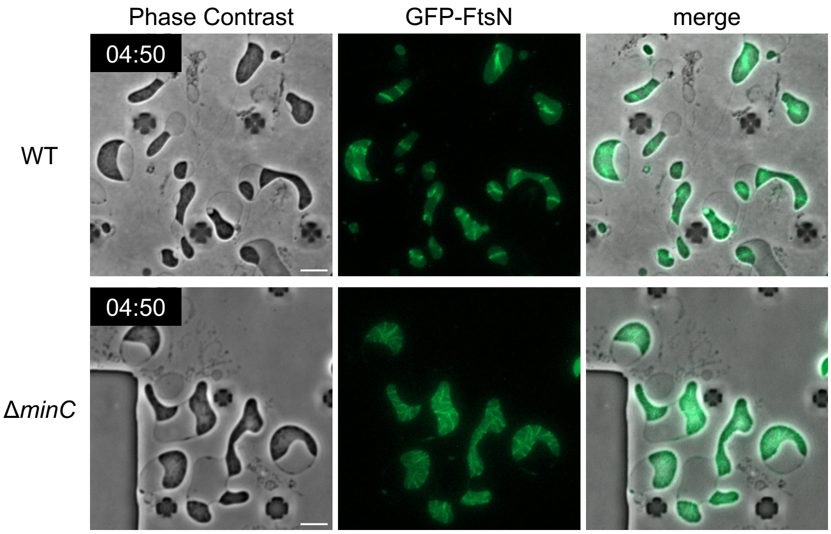

Supplementary Fig. 12. GFP-FtsN in Δ*minC* L-form cells
Time-lapse images of WT (BW25113) and RU2462 (Δ*minC*) cells carrying pRU2281. Expression of GFP-FtsN was induced by 100 μM IPTG. Cells were grown in NB/MSM medium containing Fos and IPTG under anaerobic conditions. Scale bar: 5 μm.

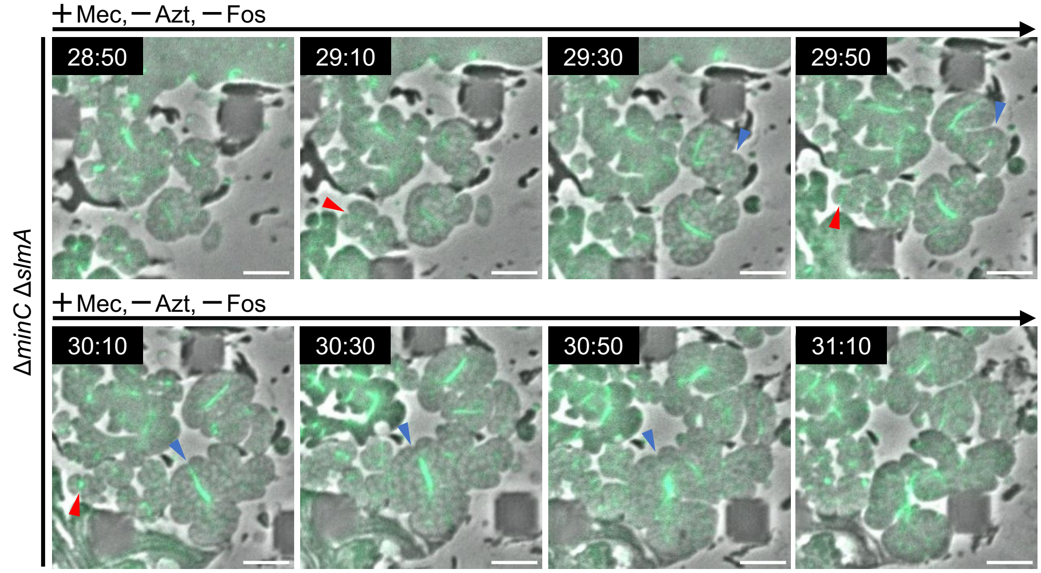

Supplementary Fig. 13. ZapA in Δ*minC* Δ*slmA* L-form cells with Fos, Mec and Azt.
Time-lapse images of RU2409 (*zapA-sfGFP hupB-mCherry* Δ*minC* Δ*slmA::kan*) cells. Cells were grown in NB/MSM medium containing Fos, Mec and Azt under anaerobic conditions. After 12h, Fos and Azt were removed from NB/MSM medium. Red arrows indicate typical cell division sites while blue arrows indicate the cell division site of cells that converted SWD cells to L-form cells. Scale bar: 5 µm.

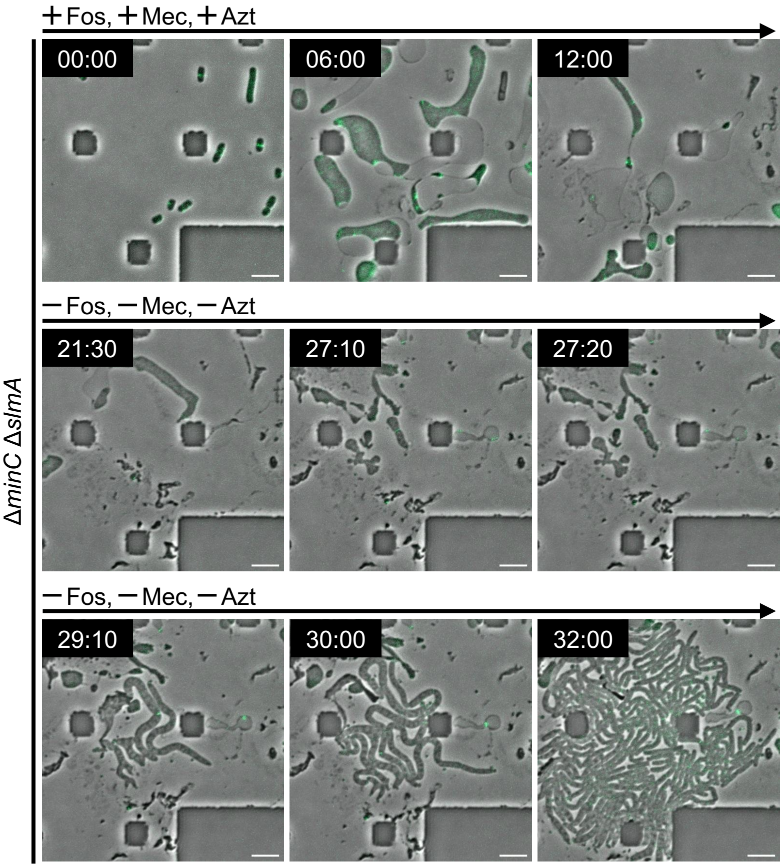

Supplementary Fig. 14. Conversions between L-form and rod-shaped cells with Fos, Mec and Azt in Δ*minC* Δ*slmA* cells.
Time-lapse images of RU2409 (*zapA-sfGFP hupB-mCherry* Δ*minC* Δ*slmA::kan*) cells. Cells were grown in NB/MSM medium containing Fos, Mec and Azt under anaerobic conditions. After 12h, Fos, Mec and Azt were removed from NB/MSM medium. Scale bar: 5 μm.

a MinC-N terminal Domain

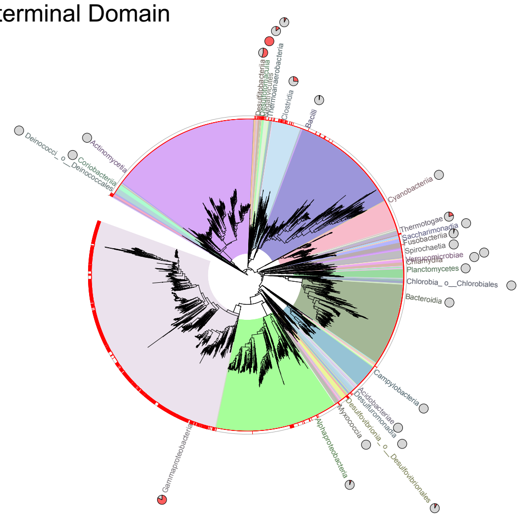

b MinC-C terminal Domain

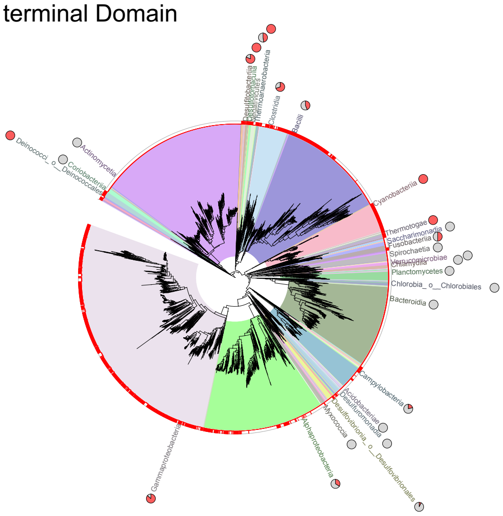

c SlmA

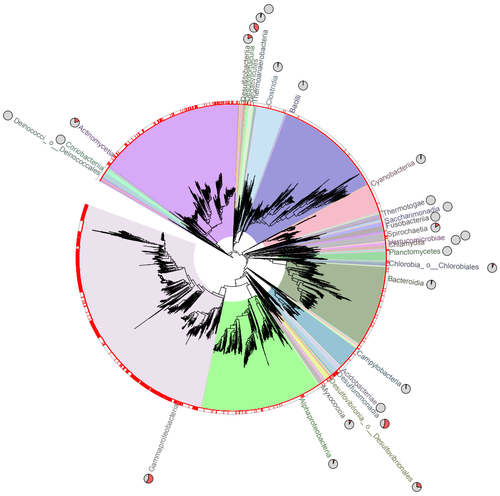

Supplementary Fig. 15. Distribution of *minC* and *slmA* genes in prokaryotes.
Phylogenetic distribution of MinC-N terminal Domain (a), MinC-C terminal Domain (b), and SlmA (c) proteins in 4,326 bacterial genomes. Red bars outside the phylogenetic tree indicate that each genome has that protein. The pie charts show, for each class, what percentage of genomes belonging to that class conserves that protein.

UNCROPPED IMAGES BEHIND IMMUNOBLOTS

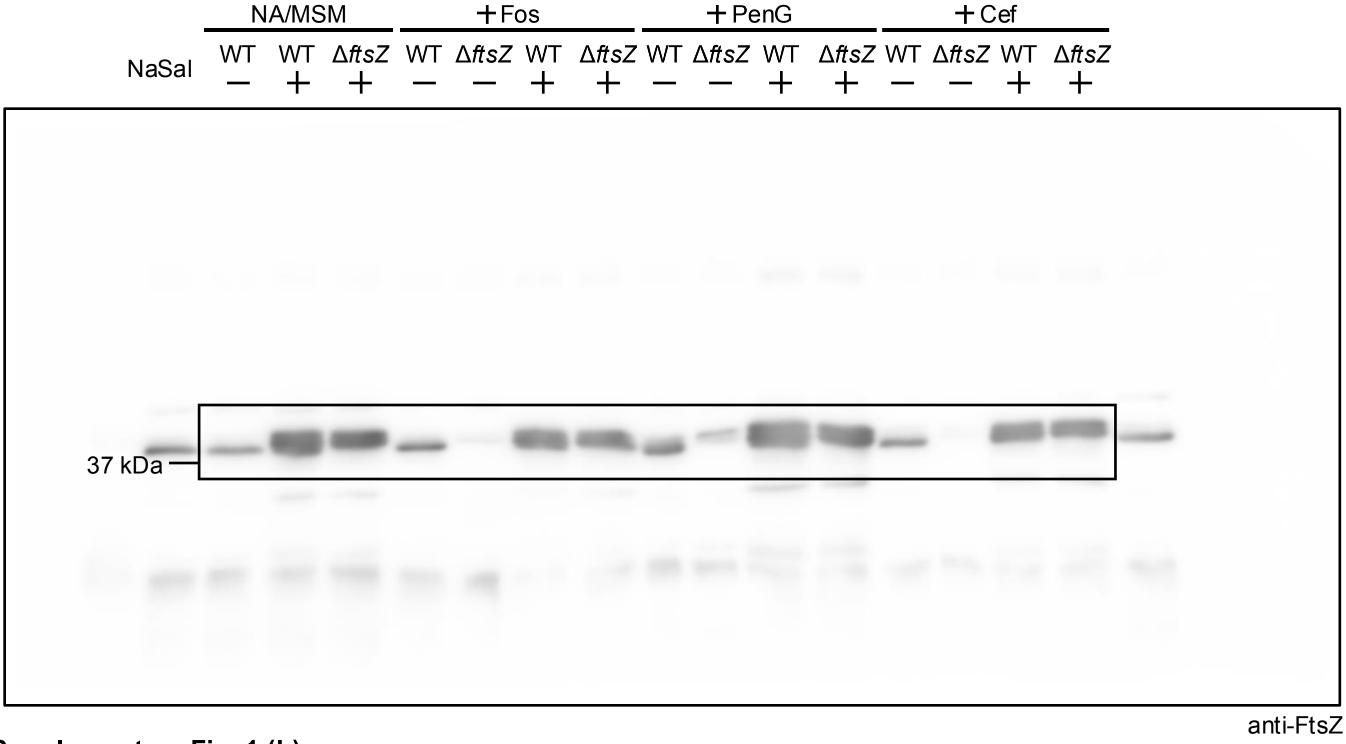

Supplementary Fig. 1 (b)
