# Supplementary material for: Septal wall synthesis is sufficient to change ameba-like cells into uniform oval-shaped cells in Escherichia coli L-forms
Source: Commun Biol. 2024 Nov 26;7:1569. doi: 10.1038/s42003-024-07279-y (PMC11589767; doi:10.1038/s42003-024-07279-y)
Supplement: Supplementary file 2 — Description of Additional Supplementary File [file 42003_2024_7279_MOESM2_ESM.pdf]

# Description of additional supplementary files

**File name:** Supplementary Data

**Description:** Contains source data for Fig. 5b in main body of manuscript.

**File name: Supplementary Movie 1**

**Description: Time-lapse movie of BW25113 in the presence of Fos.**

Time-lapse movie of the interconversion process between walled cells and L-form cells. BW25113 cells were grown in NB/MSM medium containing Fos under anaerobic conditions. After 12h, Fos was removed from NB/MSM medium. Images were taken every 10 min. Scale bar: 5 μm.

**File name: Supplementary Movie 2**

**Description: Time-lapse movie of Δ*ftsZ* cells in the presence of Fos.**

Time-lapse movie of the interconversion process between walled cells and L-form cells. RU2055 cells (Δ*ftsZ::kan* ) were grown in NB/MSM medium containing Fos in the absence of NaSal under anaerobic conditions. After 12h, Fos was removed from NB/MSM medium. Images were taken every 10 min. Scale bar: 5 μm.

**File name: Supplementary Movie 3**

**Description: Time-lapse movie of BW25113 in the presence of PenG.**

Time-lapse movie of the interconversion process between walled cells and L-form cells. BW25113 cells were grown in NB/MSM medium containing PenG under anaerobic conditions. After 12h, PenG was removed from NB/MSM medium. Images were taken every 10 min. Scale bar: 5 μm.

**File name: Supplementary Movie 4**

**Description: Time-lapse movie of Δ*ftsZ* cells in the presence of PenG.**

Time-lapse movie of the interconversion process between walled cells and L-form cells. RU2055 cells (Δ*ftsZ::kan* ) were grown in NB/MSM medium containing PenG in the absence of NaSal under anaerobic conditions. After 12h, PenG was removed from NB/MSM medium. Images were taken every 10 min. Scale bar: 5 μm.

**File name: Supplementary Movie 5**

**Description: Time-lapse movie of BW25113 and in the presence of Cef.**

Time-lapse movie of the interconversion process between walled cells and L-form cells. BW25113 cells were grown in NB/MSM medium containing Cef under anaerobic conditions. After 12h, Cef was removed from NB/MSM medium. Images were taken every 10 min. Scale bar: 5 μm.

**File name: Supplementary Movie 6**

**Description: Time-lapse movie of Δ*ftsZ* cells and in the presence of Cef.**

Time-lapse movie of the interconversion process between walled cells and L-form cells. RU2055 cells (Δ*ftsZ::kan* ) were grown in NB/MSM medium containing Cef in the absence of NaSal under anaerobic conditions. After 12h, Cef was removed from NB/MSM medium. Images were taken every 10 min. Scale bar: 5 μm.

**File name: Supplementary Movie 7**

**Description: Time-lapse movie of cell division of L-form cells without medium flow rate in the presence of Fos.**

Time-lapse movie of the cell division process in L-form cells without medium flow rate. RU1125 cells (*zapA-sfGFP::cat*) were grown in NB/MSM medium containing Fos under anaerobic conditions. After cell loading using NB/MSM medium containing Fos, the flow rate was set to 0 μL/hr. Images were taken every 10 min. Red arrows indicated cells that divided independently of medium flow. Scale bar: 5 μm.

**File name: Supplementary Movie 8**

**Description: Time-lapse movie of ZapA-GFP cells in the presence of Fos.**

Time-lapse movie of the interconversion process between walled cells and L-form cells. RU1125 cells (*zapA-sfGFP::cat*) were grown in NB/MSM medium containing Fos under anaerobic conditions. After 12h, Fos was removed from NB/MSM medium. Images were taken every 10 min. Scale bar: 5 μm.

**File name: Supplementary Movie 9**

**Description: Time-lapse movie of Δ*ftsZ* ZapA-GFP cells in the presence of Fos.**

Time-lapse movie of Z-ring reconstruction process in L-form cells. RU2057 cells (Δ*ftsZ::kan zapA-sfGFP*) were grown in NB/MSM medium containing Fos under anaerobic conditions. After 12h, 10 μM NaSal was added in NB/MSM medium. Images were taken every 10 min. Scale bar: 5 μm.

**File name: Supplementary Movie 10**

**Description: ZapA-GFP in L-forms.**

The z-stacks of deconvolved images of ZapA-GFP in L-forms.

**File name: Supplementary Movie 11**

**Description: Time-lapse movie of ZapA-GFP cells in the presence of Mec or Azt.**

Time-lapse movie of the morphological change process containing only Mec or Azt. RU1125 cells (*zapA-sfGFP::cat*) were grown in NB/MSM medium containing Mec (left panel) or Azt (right panel) under anaerobic conditions. Images were taken every 10 min. Scale bar: 5 μm.

**File name: Supplementary Movie 12**

**Description: Time-lapse movie of ZapA-GFP cells in the presence of Fos, Mec and Azt.**

Time-lapse movie of the interconversion process between walled cells and L-form cells. RU1125 cells (*zapA-sfGFP::cat*) were grown in NB/MSM medium containing Fos, Mec and Azt under anaerobic conditions. After 12h, Fos (left panel), Mec (center panel) and Azt (right panel) was removed from NB/MSM medium. Images were taken every 10 min. Scale bar: 5 μm.

**File name: Supplementary Movie 13**

**Description: Time-lapse movie of ZapA-GFP cells conversion to SWE cells from L-form cells.**

Time-lapse movie showing the process of conversion to L-form cells and SWE cells. RU1125 cells (*zapA-sfGFP::cat*) were grown in NB/MSM medium containing Fos, Mec and Azt under anaerobic conditions. After 12h, Fos and Mec were removed from NB/MSM medium. Images were taken every 10 min. Scale bar: 5 μm.

**File name: Supplementary Movie 14**

**Description: Time-lapse movie of ZapA-GFP cells conversion to SWD cells from L-form cells.**

Time-lapse movie showing the process of conversion to L-form cells and SWD cells. RU1125 cells (*zapA-sfGFP::cat*) were grown in NB/MSM medium containing Fos, Mec and Azt under anaerobic conditions. After 12h, Fos and Azt were removed from NB/MSM medium. Images were taken every 10 min. Scale bar: 5 μm.

**File name: Supplementary Movie 15**

**Description: Time-lapse movie of GFP-FtsN$^{SPOR}$ expressed cells conversion to SWD cells from L-form cells.**

Time-lapse movie showing the process of conversion to L-form cells and SWD cells.

RU2452 (*zapA-mCherry*) cells producing GFP-FtsN$^{SPOR}$ grown in NB/MSM medium containing Fos, Mec and Azt. After 12 h, Fos and Azt were removed from the NB/MSM medium. After

16h, the growth conditions were changed from anaerobic to aerobic. Images were taken every 10 min. Scale bar: 5 μm

**File name: Supplementary Movie 16**

**Description: Time-lapse movie of Δ*minC* Δ*slmA* cells in the presence of Mec.**

Time-lapse movie of the interconversion process between walled cells and L-form cells. RU2409 cells (*zapA-sfGFP hupB-mCherry* Δ*minC* Δ*slmA::kan*) were grown in NB/MSM medium under anaerobic conditions containing Fos, Mec and Azt. After 12h, Fos and Azt were removed from NB/MSM medium. Images were taken every 10 min. Scale bar: 5 μm.

**File name: Supplementary Movie 17**

**Description: Time-lapse movie of Δ*minC* Δ*slmA* cells conversion to walled cells from L-form cells.**

Time-lapse movie showing the process of conversion to L-form cells and SWD cells. RU2409 cells (*zapA-sfGFP hupB-mCherry* Δ*minC* Δ*slmA::kan*) were grown in NB/MSM medium under anaerobic conditions containing Fos, Mec and Azt. After 12h, Fos, Mec and Azt were removed from NB/MSM medium. Images were taken every 10 min. Scale bar: 5 μm.
